# The role of antibiotic exposure and the effects of breastmilk and human milk feeding on the developing infant gut microbiome

**DOI:** 10.3389/fpubh.2024.1408246

**Published:** 2024-06-06

**Authors:** Meredith Brockway

**Affiliations:** ^1^Faculty of Nursing, University of Calgary, Calgary, AB, Canada; ^2^Alberta Children’s Hospital Research Institute, Cumming School of Medicine, University of Calgary, Calgary, AB, Canada

**Keywords:** breastfeeding, human milk, donor human milk, microbiome, antibiotics, early life exposure

## Abstract

The World Health Organization (WHO) recommends exclusive breastfeeding for the first 6 months of life followed by complementary foods and sustained breastfeeding for at least 2 years, underscoring its pivotal role in reducing infant mortality and preventing various illnesses. This perspective delves into the intricate relationship between breastfeeding practices, early life antibiotic exposure, and infant gut microbiome development, highlighting their profound influence on child health outcomes. Antibiotics are extensively prescribed during pregnancy and childhood, disrupting the microbiome, and are related to increased risks of allergies, obesity, and neurodevelopmental disorders. Breastfeeding is a significant determinant of a healthier gut microbiome, characterized by higher levels of beneficial bacteria such as *Bifidobacterium* and lower levels of potential pathogens. Despite widespread recognition of the benefits of breastfeeding, gaps persist in healthcare practices and support mechanisms, exacerbating challenges faced by breastfeeding families. This highlights the pressing need for comprehensive research encompassing breastfeeding behaviors, human milk intake, and their impact on infant health outcomes. Additionally, promoting awareness among healthcare providers and families regarding the detrimental effects of unnecessary formula supplementation could facilitate informed decision-making and bolster exclusive breastfeeding rates. Moreover, donor human milk (DHM) is a promising alternative to formula, potentially mitigating disruptions to the infant gut microbiome after antibiotic exposure. Overall, prioritizing breastfeeding support interventions and bridging research gaps are essential steps towards improving child health outcomes on a global scale.

## Introduction

The World Health Organization (WHO) recommends exclusive breastfeeding for the first 6 months of life followed by the introduction of complementary foods and sustained breastfeeding for 2 years and beyond (1). It is estimated that scaling up rates of optimal breastfeeding can prevent 823,000 child deaths globally per year (2). Infants who are breastfed have reduced rates of acute infection, asthma, obesity, diabetes, lower and upper respiratory illnesses, and acute otitis media (ear infections) (2). Unlike formula, the composition of human milk is dynamic, and it changes throughout a feed, throughout the day, and across the lactation stage (3, 4). While breastmilk provides a complete source of macro- and micro-nutrients for full-term infants, it also contains many non-nutritive bioactive components, such as hormones, immunoglobulins, growth factors, cytokines, microbes, metabolites, and human milk oligosaccharides that impact infant health (5). Many of these bioactive components directly influence the developing infant microbiome. In fact, infant feeding method, particularly breastfeeding and human milk feeding, is one of the most influential aspects on the developing infant microbiome (6). As such, promoting and protecting breastfeeding and offering exclusive human milk diets, even for full-term infants, may help to mitigate potential microbiome mediated risks to child health and development.

### Antibiotic use and exposure in perinatal and pediatric populations

Antibiotics are the most prescribed medication during pregnancy and childhood, accounting for approximately 80% of prescriptions (7–9). A Swedish population based-cohort study found that across 125,106 pregnancies, 25.9% of mothers received antibiotics during pregnancy and over 40% of children were prescribed antibiotics in their first 2 years of life (10). Similarly in Canada, an examination of the Quebec Pregnancy Registry found that 24.5% of mothers used antibiotics during pregnancy. Antenatally (during labour), approximately 15–40% of mothers are exposed to antibiotics for Group B *Streptococcus* prophylaxis (11).

In the infant and pediatric populations from high income setting, antibiotic prescription rates in acute care exceed 35%, many in the first 30 days of life (9), whereas antibiotic prescription rates exceed 45% in outpatient care (12). As such, close to half of all children will be exposed to antibiotics during critical windows of development. While antibiotics are an important anti-infective agent, their use over the past several decades has become wide spread, often being used as a prophylaxis to prevent infection (9). It was previously believed that antibiotic exposure posed minimal risk, however as a medical community, we have come to learn that exposure to antibiotics is a concern from a resistance perspective as well as the impact that antibiotics can have on commensal microbiota in the human body (13).

### Early life antibiotic exposure and child health outcomes

Early life antibiotic exposure has been associated with poorer health outcomes for children and adults. A systematic review of 160 studies examining outcomes of over 22 million children found that children who were exposed to antibiotics early in life have significantly increased risks of developing atopy and allergy, obesity and overweight, and neurodevelopmental disorders such as autism and attention deficit hyperactivity disorder (14). Additionally, it is likely that there is a dose response with increased antibiotic exposure yielding an increased risk in developing adverse health outcomes (10). It is well supported that many of these disorders may be related to the disruptions that antibiotics create in the infant microbiome during critical windows of child development.

The first 1,000 days of life, from conception to 2 years of age, is the critical period when the bacterial composition of the infant’s gut microbiome helps to shape its developing immune system (15). A dysbiotic, or imbalanced, gut microbiome during critical windows of infant development in the first 1,000 days can have long-term negative consequences on child health outcomes such as metabolic diseases, asthma and allergy, and altered neurodevelopment (16). Ideally, the infant gut microbiome is established when the mother transmits microbes to their infant during vaginal delivery, followed by exclusive breastfeeding and no antibiotic treatment (15). However, more than half of full-term infants experience deviations from this cascade, including interventions such as c-section delivery (17), formula feeding (18, 19), and antibiotic exposure (7–9) which can adversely impact their developing microbiome (20).

Exposure to antibiotics in the perinatal and neonatal period significantly alters the developing infant gut microbiome, leading to a disrupted or dysbiotic state (21). Antibiotic use during pregnancy significantly disrupts the gut and vaginal microbiomes of mothers, reducing the α diversity of the bacteria that infants are exposed to when they are born vaginally, thereby impacting the initial colonization of the infant microbiome (22). Intrapartum antibiotic exposure, usually for Group B *Streptococcus* prophylaxis, is associated with a lower relative concentration of Bifidobacteriaceae, and increased relative abundance of *Proteobacteria* in the gut microbiome of exposed infants compared with those who are not exposed (21). Exposure to antibiotics during infancy significantly alters the composition of the microbiome in the developing gut, resulting in reduced levels of essential anaerobic bacteria like *Bifidobacteria*, *Lactobacilli*, and *Bacteroides*, as well as diminished populations of butyrate-producing families such as Bifidobacteriaceae, Bacteroidaceae, and Eubacteriaceae (23). Additionally, maternal use of antibiotics while breastfeeding significantly alters the breastmilk microbiome and the antibiotics can pass through the breastmilk to impact the infant’s developing microbiome as well (23, 24).

Evidence consistently suggests that antibiotic exposure during pregnancy, breastfeeding and in the neonatal period, reduces colonization and abundance of important commensal bacteria such as *Bifidobacterium* (25). *Bifidobacteria* are important early colonizers of the gut because they are primary consumers of prebiotic oligosaccharides in human milk, they crowd out pathogenic bacteria, and their presence is associated with decreased risk of atopy and other childhood diseases (26).

### Can breast milk and human milk feeding help to recover the infant gut microbiome?

After birth, breastfeeding is one of the most influential factors on the developing infant gut microbiome. Breastfed infants consistently demonstrate higher levels of *Bifidobacterium* compared to infants who receive formula (27, 28). Compared to exclusively formula fed infants, exclusively breastfed infants have lower α diversity which remains stable over the first 3 months of life and increases at 6 months (28). Conversely, formula fed infants demonstrate increased α diversity early in life, which is reflective of the gut microbiome in older children (28). Further, breastfed infants have increased levels of commensal bacteria such as *lactobacilli* and *enterococci*, and reduced levels of pathogenic bacteria such as *Clostridium perfringens*, *Klebsiella oxytoca*, and *Enterococcus faecalis* (6).

**Figure 1:**
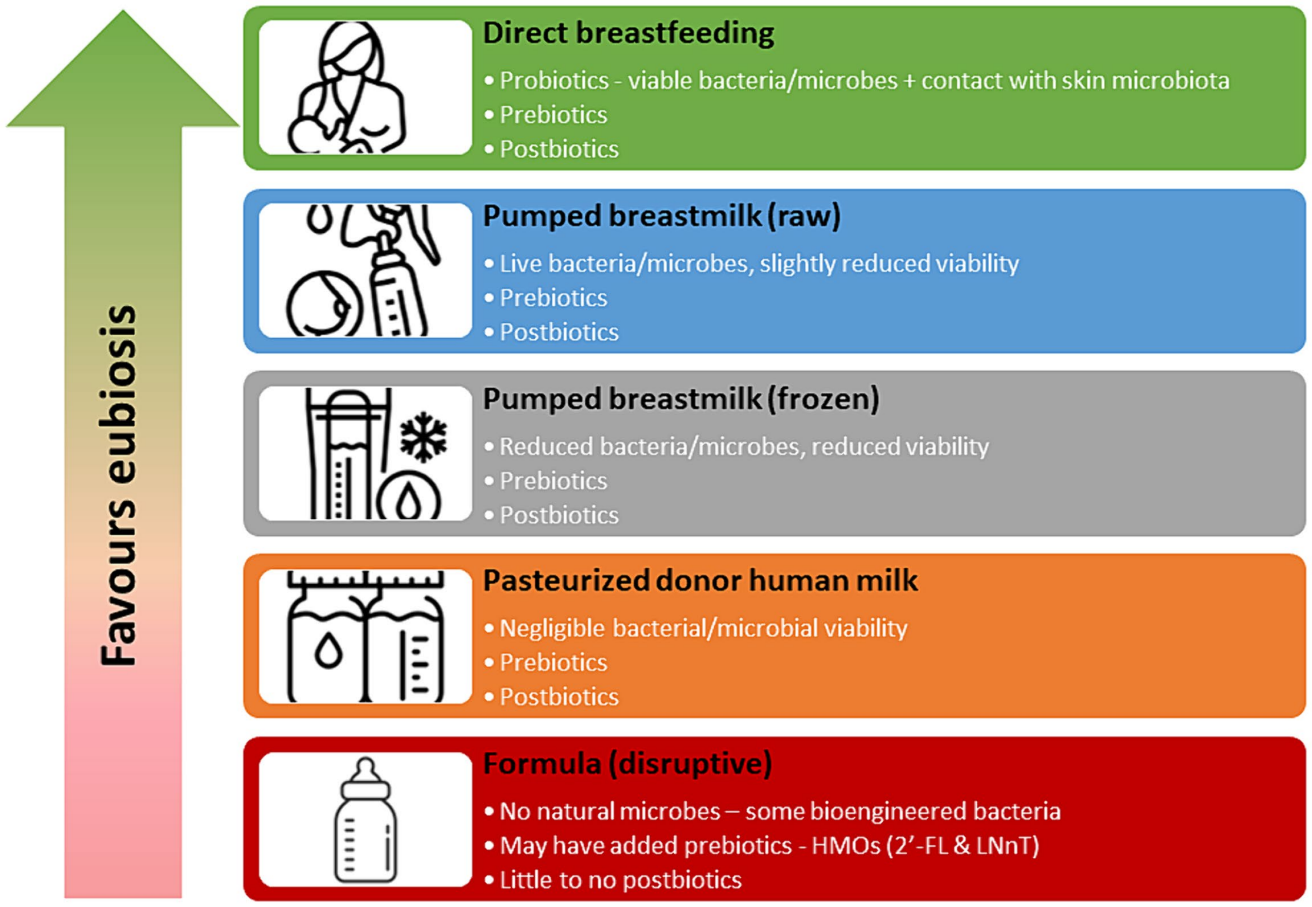
Biotic properties of human milk and formula based on mode of feeding.

Human milk contains pro-, pre- and post-biotic substances that directly impact the developing infant microbiome. Raw human milk, fed directly from the breast contains live bacteria, or probiotics, consisting of more than 800 bacterial species, that can colonize the infant gut (29). An exclusively breastfed infant consumes 1 × 10^5^ and 1 × 10^7^ bacteria per day (30), making human milk the second most important source of colonization of the infant gut microbiome, following bacterial exposure via the birth canal (29). Processing of human milk, such as decanting, storage, refrigeration, freezing and pasteurization can drastically impact the viability of probiotics in human milk (31, 32). While direct breastfeeding confers the greatest levels of probiotic exposure, previously refrigerated and frozen milk also still demonstrates viable microbial activity (Figure 1). Pasteurized donor human milk (DHM) has minimal microbial activity (32), and while not sterile, is not considered a viable source of probiotics.

Human milk also contains pre-biotics in the form of human milk oligosaccharides (HMOs) which are the third most prevalent component in human milk (33). There are over 150 different HMO structures in human milk and only about 1% are absorbed into the circulation of the infant (34, 35). The remaining 99% are believed to be metabolised by gut microbes or excreted by the infant (34). The primary role of HMOs is to serve as a pre-biotic for commensal bacteria in the infant gut (35). While HMOs do not naturally occur in formula, 2′-FL (2′-Fucosylactose) and LNnT (Lacto-N-neotetraose) are approved to be added to infant formulas in the US and Canada (35). Processing of human milk does not significantly reduce the pre-biotic activity and pasteurized DHM has similar levels of HMOs compared to raw human milk and is an excellent source of pre-biotics (36) (Figure 1).

Finally, human milk contains post-biotics, which are metabolites produced from microbial fermentation. Post-biotics in human milk consist of microbial cells, cell constituents and metabolites such as short-chain fatty acids (37). Presence of post-biotics may inhibit the growth of pathogens in the infant gut, enhance intestinal barrier function and mucosal immunity, and promote gene transcription (37). A consensus statement on the definition of post-biotics was only recently developed by the International Scientific Association of Probiotics and Prebiotics (ISAPP) in 2021 (38) and as such, rigorous research in this field is just emerging (37). However, it appears that processing of human milk has minimal impact on many post-biotic components and pasteurized DHM remains a good source (39).

Specific to antibiotic exposure, Dai et al. (40) found that any breastfeeding enriched *B. longum infantis* in the infant gut. This study determined that infants who were exposed to antibiotics and who were receiving any breastmilk at 3 months of age had a significantly reduced risk of developing asthma and that this relationship was mediated by *Bifidobacterium longum Subspecies infantis (Bifidobacterium infantis)* which is a bacterium that is dependent on human milk (40). They summarized that most species in the infant microbiome that were affected by antibiotics are responsive to breastfeeding and can be rescued to non-antibiotic levels in breastfed infants. This is one of the first studies to examine the reparative effects of breastfeeding on the microbiome of infants who are exposed to antibiotics. However, due to power limitations, they were not able to examine the effects of exclusive or direct breastfeeding on the infant microbiome.

#### Forms of breastfeeding – does it matter?

It is likely that the form of breastfeeding (exclusive breastfeeding compared to combo/mixed feeding or direct breastfeeding compared to feeding expressed milk) may have varying impacts on the infant microbiome. Exclusive breastfeeding is defined as the infant only receiving breastmilk with no other fluids or complementary foods (1) and the WHO recommends exclusive breastfeeding for the first 6 months of life. Observational evidence indicates that exposure to small amounts of formula in the first days of life can significantly impact the composition of the microbiome at 3-months of age (18). Despite exclusive breastfeeding after leaving the hospital, infants who were fed formula in hospital had lower relative abundance of *Bifidobacteriaceae* in the gut microbiome at 3 months postpartum compared to infants who were exclusively breastfed throughout (18). Exclusive breastfeeding also appears to have a recovering effect on the microbiome of infants who are born via caesarean section. Liu et al. (41) found that infants born via caesarean section who were exclusively breastfed at 6 months of life had gut microbiomes that resembled infants who were born vaginally. This association was not observed for infants who received both formula and breastmilk. Unfortunately, Liu et al. (41) did not indicate if the infants who were exclusively breastfed at 6 months had ever received formula previously.

Duration of exclusive breastfeeding also appears to have an important impact on the developing infant gut microbiome. A recent systematic review and meta-analysis demonstrated a pooled protective effect of longer duration of exclusive breastfeeding and reduced risk of developing asthma for children under 7 years of age (42). Infants who are exclusive breastfed for longer periods of time are 19% less likely to develop asthma compared to children who were exclusively breastfed for shorter periods of time. Further, comparing infants who were ever breastfed to never breastfed did not yield significant differences in asthma risk, indicating that exclusive breastfeeding, or lack of formula exposure is most impactful on infant health outcomes (42). This is an important distinction as it highlights the importance of breastfeeding exclusivity, but also the duration of exclusive breastfeeding as well. While research around exclusive breastfeeding is scant, plagued by poorly defined outcomes, and highly heterogenous, it is evident that a dose response for exclusivity and duration of exclusivity exists.

Mode of breastfeeding also appears to play an important role in infant health, likely mediated via the developing infant gut microbiome. Observational evidence from the CHILD study indicates that infants who are directly fed from the breast compared to infants who are fed their mother’s expressed milk have significantly reduced risk of asthma diagnoses at 3 years of age (43). Further, milk from parents who express rather than directly breastfeed demonstrates lower bacterial richness and this richness also differs between milk that is pumped compared to milk that is manually expressed (44). It is likely that the varied richness of the milk microbiota directly informs the colonization of the infant gut, as up to 30% of the infant microbiome is derived from breastmilk intake (45). Despite mode of breastfeeding, it is important to note that both direct and expressed feeding of breastmilk confers a significant risk reduction of asthma diagnoses compared to formula feeding (43).

It is evident that gut microbiome signatures are heavily influenced by infant feeding type (28, 46) and breastfeeding can improve gut microbiota composition in infants who experience adverse early life exposures (47). It also appears that exclusive breastfeeding and direct breastfeeding may provide enhanced protection for infants who experience adverse early life disruptions to their microbiome such as antibiotic exposure. However, future research in child health needs to carefully measure exclusivity of breastfeeding, considering if there has been any previous formula exposure and how the breastmilk is being provided to the infant.

## Clinical and research considerations

Promotion and protection of breastfeeding and exclusive human milk feeding is a public health intervention that is affordable and effective in improving child health outcomes on a global scale (2). However, there still exist some gaps in the evidence and many areas of healthcare lack evidence-based practice strategies to support breastfeeding families. Further, as evidence of breastfeeding benefits have started to accumulate over the past 20–30 years, public and health discourse around infant feeding has increasingly become very pro-breastfeeding and mothers are under intense social pressure to exclusively breastfeed their infants (48, 49). Yet, society provides minimal support or choice of supplementation when breastfeeding is not possible or does not go as planned.

### Considerations for research

Breastfeeding and human milk intake has long been under reported and not accurately measured in child health research. Further, breastfeeding evidence is predominantly limited to observational studies exploring associations between breastfeeding and infant health outcomes, which have been critiqued for their risk of confounding (48). While the WHO and most healthcare agencies recommend exclusive breastfeeding for the first 6 months of life (1), rigorous evidence to support these guidelines is still lacking. Increasingly, we are observing the profound impact and protective effect that breastfeeding and human milk feeding has on infant development and child health outcomes (2). It is likely that this relationship is mediated by the role human milk has on the developing gut microbiome in infants (46). However, conclusive evidence about this relationship is lacking. Future research needs to include careful measurement of breastfeeding behaviors and human milk intake. Collecting data on exclusivity of breastfeeding, mode of breastfeeding, amount of supplementation, and type of supplementation will provide much stronger evidence around the relationship between infant feeding and infant health. In addition, prioritizing the relationship between infant feeding and the developing infant microbiome in research will help to provide a better understanding of the mechanism and dose response of human milk feeding on the developing infant microbiome.

### Provider education

There have been many calls to action to further protect and promote breastfeeding. These have been relatively successful in increasing breastfeeding initiation rates to >90% in Canada (50), and to >85% in the United States (51). However, over 60% of mothers do not meet their breastfeeding goals (52) and exclusive breastfeeding rates fall well below the recommended guidelines (50, 51). The most common reasons for formula supplementation in the early days of life are related to medical indications, such as hypoglycemia, weight loss, or jaundice; parental request or preference; and lactation management issues, such as poor latch or perceived insufficient milk supply (53). In addition, it is still common for healthcare providers to defer to formula without sufficient medical indication or providing adequate support to breastfeeding families (53, 54). Providing mandatory education for healthcare providers who work with families in the perinatal setting is an evidence-based strategy that helps to improve breastfeeding rates (2, 54, 55). Additionally, raising awareness among healthcare providers and families around the unethical marketing of breastmilk substitutes from formula companies may help to enhance critical thinking around unnecessary formula supplementation and increase capacity for informed decision making with infant feeding (56).

### Supplementation options

Exclusive breastfeeding rates still lag far behind recommended guidelines (50, 51). It is estimated in Canada and the United States, 40–60% of full-term infants receive at least one bottle of formula supplementation in their first week of life (18, 19, 51). Not only does this disrupt the infant microbiome (18), but it also disrupts the establishment of breastfeeding and leads to lower breastfeeding exclusivity and duration rates (57, 58). Donor human milk (DHM) is likely a superior alternative to formula when supplementation is required because it allows infants who are supplemented to continue to be exclusively fed human milk. Through its various biotic properties, DHM minimizes perturbations to the gut microbiome in infants who experience adverse early-life exposures (59, 60). As such, there are likely profound differences in the impact of formula supplementation compared to DHM supplementation on the developing infant gut microbiome (36, 61). Additionally, evidence indicates that mothers who supplement with DHM compared to formula may be more likely to continue exclusively breastfeeding their infants at 6 months (62). However, there is minimal research on the impact of DHM as a supplementation option for the full-term population and on the full-term infant gut microbiome (63). While DHM is the accepted standard of practice for preterm infants when supplementation is indicated in clinical settings, little is known about DHM use in the full-term infant population. Research in this area is laden with opportunity to establish causal relationships between DHM supplementation (exclusive human milk feeding) compared to formula supplementation on the infant gut microbiome. Randomization of infants to receive DHM instead of formula is still an ethical practice because DHM supplementation is not standard practice in the full-term population. DHM is a viable and feasible alternative to formula supplementation that should be considered for full-term infants who experience early life perturbations to their microbiome.

## Conclusion

The interplay between breastfeeding, early life antibiotic exposure, and infant gut microbiome development underscores the critical importance of promoting and protecting breastfeeding and human milk feeding for optimal child health outcomes. While lifesaving, unnecessary antibiotic exposure and overuse is a significant concern and may pose long-term risks to child health. Breastfeeding is associated with a myriad of benefits, including a healthier gut microbiome and reduced risks of infections and diseases and may help overcome some of the perturbations to the microbiome from antibiotic exposure. However, current breastfeeding rates lag behind WHO recommendations. Addressing these challenges requires a multi-faceted approach, including comprehensive research to elucidate the mechanisms underlying breastfeeding’s protective effects and the impact of interventions like DHM supplementation. Additionally, healthcare providers must prioritize breastfeeding support and education to empower families in making informed decisions regarding infant feeding. By bridging research gaps, enhancing breastfeeding support mechanisms, and raising awareness about the advantages of exclusive breastfeeding and DHM supplementation, we can strive towards improving child health outcomes and fostering a healthier future generation. Ultimately, investing in breastfeeding promotion and support initiatives is a cost-effective public health intervention that deserves commitment and dedication from policymakers, healthcare providers, and society as a whole.

## Data availability statement

The original contributions presented in the study are included in the article/supplementary material, further inquiries can be directed to the corresponding author.

## Author contributions

MB: Conceptualization, Investigation, Methodology, Resources, Supervision, Visualization, Writing – original draft, Writing – review & editing.
